# Ectopic Fat Accumulation in Distinct Insulin Resistant Phenotypes; Targets for Personalized Nutritional Interventions

**DOI:** 10.3389/fnut.2018.00077

**Published:** 2018-09-04

**Authors:** Inez Trouwborst, Suzanne M. Bowser, Gijs H. Goossens, Ellen E. Blaak

**Affiliations:** Department of Human Biology, NUTRIM School of Nutrition and Translational Research in Metabolism, Maastricht University Medical Center+, Maastricht, Netherlands

**Keywords:** insulin resistance, ectopic fat, personalized nutrition, type 2 diabetes mellitus, metabolic phenotype

## Abstract

Cardiometabolic diseases are one of the leading causes for disability and mortality in the Western world. The prevalence of these chronic diseases is expected to rise even further in the next decades. Insulin resistance (IR) and related metabolic disturbances are linked to ectopic fat deposition, which is the storage of excess lipids in metabolic organs such as liver and muscle. Notably, a vicious circle exists between IR and ectopic fat, together increasing the risk for the development of cardiometabolic diseases. Nutrition is a key-determining factor for both IR and ectopic fat deposition. The macronutrient composition of the diet may impact metabolic processes related to ectopic fat accumulation and IR. Interestingly, however, the metabolic phenotype of an individual may determine the response to a certain diet. Therefore, population-based nutritional interventions may not always lead to the most optimal (cardiometabolic) outcomes at the individual level, and differences in the metabolic phenotype may underlie conflicting findings related to IR and ectopic fat in dietary intervention studies. Detailed metabolic phenotyping will help to better understand the complex relationship between diet and metabolic regulation, and to optimize intervention outcomes. A subgroup-based approach that integrates, among others, tissue-specific IR, cardiometabolic parameters, anthropometrics, gut microbiota, age, sex, ethnicity, and psychological factors may thereby increase the efficacy of dietary interventions. Nevertheless, the implementation of more personalized nutrition may be complex, costly, and time consuming. Future studies are urgently warranted to obtain insight into a more personalized approach to nutritional interventions, taking into account the metabolic phenotype to ultimately improve insulin sensitivity and reduce the risk for cardiometabolic diseases.

## Introduction

Cardiometabolic diseases such as obesity, diabetes mellitus type 2 (T2DM), and cardiovascular diseases are the leading causes of death and disability in the Western world. In 2014, T2DM affected about 422 million adults worldwide, and its prevalence is expected to rise even further in the next decades (1), representing a large burden for society. Although different etiologies precede the development of cardiometabolic diseases, insulin resistance (IR) is a major contributing risk factor, and is therefore a relevant target for preventive healthcare.

IR represents a physiological state in which the action of the hormone insulin is impaired. As a consequence, the body is not effectively able to adapt to its metabolic or energy demands, also described as metabolic inflexibility (2, 3). IR can develop simultaneously in multiple organs and the IR severity may vary between different organs. More specifically, the regulation of metabolic processes such as glucose uptake and oxidation, glycogen synthesis and breakdown, lipid storage, and lipolysis may be disturbed. A wide range of literature has linked these metabolic disturbances to accumulation of excess lipids in organs such as the liver, skeletal muscle, pancreas, and heart, also referred to as ectopic fat deposition (4, 5). The link between ectopic fat, IR and metabolic inflexibility, however, is multifaceted. The type of tissue (e.g., liver or skeletal muscle), the accumulation of lipids (e.g., triacylglcyerol, diacylglycerol) and lipid metabolites (e.g., ceramides and acylcarnitines) as well as composition, turnover, and localization of lipids have all been shown to play important roles in the metabolic consequences of ectopic fat accumulation (6, 7). More globally, age, sex, lifestyle, and (epi)genetics, as well as microbial composition and functionality, may also play an important role in ectopic fat accumulation (5, 8, 9). These factors impact the development of distinct IR phenotypes, and should be considered when targeting ectopic fat and IR.

Diet and physical activity are key modifiable risk factors that play a significant role in the development, progression and reversal of IR and ectopic fat accumulation (5, 10). Related to the diet, not only caloric intake, but also the macronutrient quantity and quality of the diet may affect insulin sensitivity, which may be mediated by changes in ectopic fat accumulation (11–14). Interestingly, however, recent evidence has shown that the metabolic phenotype of an individual seems to contribute to the inter-individual differences in response to dietary intervention (13, 15). Therefore, interventions to prevent or reverse IR may be optimized using a more subgroup-based or personalized approach.

In this review, we will provide an overview of the mechanisms that play a role in ectopic fat storage and its relationship to distinct IR phenotypes. Additionally, it will be discussed how dietary macronutrient quality and quantity may affect ectopic fat and, consequently, IR in individuals with different metabolic phenotypes.

## Ectopic fat and insulin resistance: pathophysiology and inter-relationships

### Cellular mechanism leading to ectopic fat and IR

IR conditions are often accompanied by a systemic overflow of lipids, which is mainly the result of disturbances in adipose tissue lipid handling, in combination with a chronic excess energy intake (2). Disturbances in adipose tissue function are, beside an impaired lipid storage capacity, characterized by an increased infiltration of macrophages and other immune cells leading to a state of chronic low-grade inflammation in obesity (2). Adipose tissue inflammation may induce alterations in adipose tissue metabolism (i.e., lipid metabolism/lipolysis) and the storage capacity of dietary lipid in adipose tissue (2, 16). The excessive flux of lipids toward multiple oxidative tissues, including the liver and skeletal muscle, has marked effects on lipid uptake and metabolism in these tissues, thereby contributing to the development of ectopic fat deposition and IR. Furthermore, the increased secretion of inflammatory factors by adipose tissue may lead to systemic inflammation, which in turn may affect insulin signaling and metabolism of non-adipose tissues, like the liver and skeletal muscle, contributing to the accumulation of ectopic fat and development of IR (2). The mechanisms by which ectopic fat accumulates and how this accumulation affects IR are, however, different between tissues, and has been extensively studied for the skeletal muscle (17). Importantly, the link between ectopic fat and IR is not a one-directional pathway. Accumulation of lipids within the liver, for example, may also be driven by increased *de novo* lipogenesis due to hyperinsulinemia, as present in IR conditions, and by increased NEFA supply from the adipose tissue (18, 19). Additionally, a reduced capacity to oxidize fatty acids upon increased supply in skeletal muscle (metabolic inflexibility) is often observed in obese insulin resistant conditions, which may be accompanied by mitochondrial dysfunction (2). Hence, a vicious cycle exists between the progression of IR and ectopic fat deposition. In the following sections, we will provide an overview of the mechanisms underlying the accumulation of ectopic fat in the liver and skeletal muscle and its effect on IR.

#### Disturbances in liver and skeletal muscle lipid uptake

IR individuals are characterized by an altered plasma lipid profile (20). In fasted and postprandial IR conditions, (slightly) elevated levels of non-esterified fatty acids (NEFA), very-low-density lipoprotein (VLDL)-TAG (21), and dietary-derived chylomicron-TAG (22) are observed, although data is not entirely consistent (23, 24). Notably, NEFA concentrations may not be elevated in proportion to the increased fat mass, since NEFA concentrations per unit fat mass are down regulated in individuals with excess adipose tissue mass (24). Elevated levels of circulating TAG concentrations may be attributed to an increased VLDL-TAG production by the liver or reduced clearance of TAG by adipose tissue (2). Indeed, the removal of TAG across adipose tissue was found to be impaired in obesity, insulin resistance and T2DM due to a reduced insulin-mediated stimulation of lipoprotein lipase (LPL) activity (23, 25–29), suggesting less efficient removal of dietary lipids by adipose tissue in these subjects. Notably, LPL in AT macrophages has been found to increase AT lipid storage in obese mice (30, 31). In line, studies in high-fat-diet-fed mice showed local lipid fluxes are central regulators of AT macrophage recruitment and that once recruited these macrophages can buffer local increases in lipid concentrations (30). These data suggest that AT macrophages may compensate for the insufficient lipid storage in adipocytes in the obese insulin resistant state, reversing thereby glucose intolerance and insulin resistance.

Despite slightly elevated plasma NEFA concentrations in T2DM as compared to healthy control subjects, a similar postprandial NEFA uptake in skeletal muscle was observed in both groups (32). Likewise, we have recently demonstrated a comparable skeletal muscle uptake of circulating NEFA in overweight and obese subjects with mild vs. more severe IR (33). Elevated plasma NEFA levels may, however, lead to increased uptake of NEFA into the liver (34). An increased supply of NEFA to the liver leads to an increased partitioning of NEFA toward VLDL-TAG synthesis, and may be accompanied by a decreased insulin-mediated suppression of VLDL-TAG synthesis (35). Both processes contribute to elevated postprandial VLDL-TAG levels in IR. Interestingly, skeletal muscle extraction of VLDL-TAG in postprandial conditions is higher in more severe vs. mild IR individuals, despite similar VLDL-TAG supply (7, 33). In accordance with these findings, IR subjects show higher skeletal muscle VLDL-TAG extraction than normal glucose tolerant (NGT) individuals (36). Together, these findings indicate that disturbances in lipid supply and/or uptake in both liver and skeletal muscle may contribute to the pathogenesis of ectopic fat deposition and IR.

Plasma lipids can be transported into the liver or skeletal muscle cell by receptor-mediated transport, as well as passive diffusion via the cell membrane (37). The rate of lipid uptake is dependent on the type of tissue, type, and activity of transporter proteins on the cell membrane, and is a key factor in lipid handling by the liver or skeletal muscle cells. LPL in skeletal muscle and the membrane carrier proteins (specifically CD36) and fatty acid transport proteins (FATP) in liver and skeletal muscle are, therefore, an integral part of this process. LPL is, next to its large abundance in adipose tissue, expressed in endothelial cells near skeletal muscle and is an important facilitator of chylomicron- and VLDL-TAG extraction across the endothelium into the skeletal muscle cell (38). LPL has the capacity to bind and hydrolyze TAG on the surface of the capillary lumen, thereby releasing NEFA which can be taken up by the tissue (39). FATPs are involved in the transport of long-chain fatty acids across the membrane. Different members of the FATP family are expressed in the liver (FATP3,5) and skeletal muscle (FATP3,4), but may have similar properties related to lipid uptake (40). CD36 is a well-characterized protein that is also responsible for the uptake of a variety of lipids in the cell. Expression of FATPs and CD36 in liver and skeletal muscle is often dependent on the metabolic condition. Overexpression of these transport proteins is often seen in response to hyperinsulinemia, and may lead to an increased uptake of lipids in IR conditions, which in turn contributes to ectopic fat accumulation in these tissues and associated metabolic impairments (38, 41–44). Interestingly, this may not be the case for LPL in skeletal muscle since insulin seems to stimulate LPL activity in adipose tissue, but not in skeletal muscle (45).

Altogether, human data suggest that merely TAG rather than NEFA uptake into the skeletal muscle may be a putative target in the prevention and/or treatment of ectopic fat deposition and IR in individuals with an impaired oxidative capacity. The role of disturbances in lipid uptake by the liver in relation to liver fat accumulation and hepatic IR is less established.

#### Disturbances in intramyocellular lipid metabolism

In addition to lipid uptake, insulin sensitivity is strongly related to the oxidative capacity of key metabolic organs such as the liver and the skeletal muscle. The high oxidative capacity represents the ability of the body to adapt to the appropriate metabolic condition, also described as metabolic flexibility (46). Following uptake from the circulation, lipids can either be oxidized or stored for later use. In healthy, insulin sensitive individuals, lipid accumulation within the cell is limited, albeit not absent, as an elevated influx of lipids into the cell triggers the conversion of lipids to long-chain fatty acyl-CoA (LC-CoA), which is transported into the mitochondria and, consequently, degraded by beta-oxidation.

In contrast, IR conditions are characterized by disturbed skeletal muscle lipid metabolism (2). In obese individuals with more severe as compared to mild IR the following has been reported: a higher saturation of skeletal muscle NEFA and DAG (in particular membrane-bound DAG), a tendency toward a lower fractional synthesis of TAG, and reduced gene expression of oxidative metabolism markers (33, 36, 47). In line, in overweight and obese adults, more severe IR individuals showed lower re-esterification of intramuscular NEFA into TAG, and decreased expression of several genes encoding for proteins involved in TAG synthesis as compared to individuals with mild IR (7). Additionally, in individuals with long-term diagnosed T2DM, a blunted insulin-mediated suppression of skeletal muscle lipolysis was reported, which was related to accumulation of more saturated DAG at the skeletal muscle membrane as compared to normal glucose tolerant (NGT) individuals (47).

These disturbances reflect, at least partly, a decreased capacity of the skeletal muscle to oxidize lipids (48) and a decreased ability to switch from carbohydrate to fat oxidation (49). The decreased skeletal muscle oxidative capacity may be a result of reduced mitochondrial activity, content, and/or plasticity, leading to an imbalance between lipid supply, lipid oxidation, and conversion into TAG (50). Furthermore, decreased mitochondrial function may lead to overproduction of reactive oxygen species (ROS), which are reactive molecules and free radicals, further contributing to impaired mitochondrial function and oxidative stress (51). The reduced oxidative capacity may contribute to lipid peroxidation whereby lipotoxic compounds are formed in the skeletal muscle, including diacylglycerol (DAG) and ceramides (48, 52). DAG and ceramides may have lipotoxic effects on mitochondrial DNA, RNA, and proteins, thereby promoting a further decline in mitochondrial bioenergetics (48). In addition, DAG are responsible for the activation of protein kinase C (PKC) isoforms. PKCs are important signaling molecules involved in insulin-stimulated glucose uptake, since PKC inhibits several steps of the insulin signaling pathway (53). As a consequence, PKC activation by lipid-intermediates interferes with insulin signaling, resulting in a reduced skeletal muscle glucose uptake, as extensively reviewed elsewhere (5). The relevance of ceramide accumulation in muscle insulin resistance in humans is still unclear (17). These findings illustrate the important role of lipotoxic lipid-intermediates in the development and progression of IR in the skeletal muscle.

#### Disturbances in intrahepatocellular lipid metabolism

As discussed, lipid accumulation within the liver has been linked to IR. The mechanisms within hepatocytes that link lipid metabolism to IR are, however, less understood. It has been shown that insulin increases hepatic (*de novo)* lipogenesis, TAG storage, as well as the production of VLDL-TAG in the liver in IR conditions (17). Moreover, similar to skeletal muscle, an impaired mitochondrial function in the liver has been found in obese IR individuals (17). The link between mitochondrial dysfunction in the liver and IR may be explained by excess lipid supply, as often seen in IR conditions. The chronic increased activation of beta-oxidation due to excess lipid supply may stimulate oxidative stress and lipotoxicity, in turn resulting in impairments in mitochondrial oxidative capacity (54). In line, *in vivo* MRS measurements showed that IR-individuals exhibited lower hepatic ATP levels, which related to liver-IR and partly accounted for hepatic lipid accumulation (55). However, a causal relationship between mitochondrial function and hepatic IR has yet to be confirmed.

Impaired mitochondrial function in the liver may lead to reduced oxidation of lipids, resulting in the formation of different lipid moieties such as ceramides (54). The accumulation of both total DAG and cytosolic DAG in the liver have been linked to HOMA-IR as well as to liver-IR, as determined by insulin-induced suppression of hepatic glucose output (56, 57). Peterson et al. (58) hypothesized that DAG mediates lipid-induced IR in the liver by inhibition of insulin signaling in a similar manner as occurs in skeletal muscle. As discussed earlier, DAG may activate several PKC isoforms, thereby inhibiting several steps of the insulin signaling pathway. Furthermore, DAG accumulation in the liver could result in decreased mitochondrial function, inflammation, and increased VLDL-TAG production, which may affect IR on a local (liver-IR) and systemic level. Consequently, DAG accumulation in the liver may contribute to liver-IR and, consequently, to decreased glycogen synthesis and increased hepatic gluconeogenesis (2, 17, 58). In humans, there is currently no evidence for a relationship between ceramide accumulation and impaired hepatic insulin action (17, 56, 57). It must be noted that the number of studies related to lipid metabolism in the liver and IR are limited, studies are predominantly cross-sectional, or *in vitro* models have been used, which makes it difficult to understand the molecular mechanisms involved in the putative relationship between disturbances in hepatic lipid metabolism and liver IR.

To summarize, cellular mechanisms responsible for IR and lipid accumulation in both liver and skeletal muscle are related to altered lipid uptake into the cell as well as to an impaired intracellular regulation of lipid metabolism. Together, these impairments may cause an excess accumulation of lipotoxic metabolites if oxidative capacity is insufficient, which in turn interferes with insulin signaling.

### Ectopic fat and liver- and muscle-IR

As mentioned earlier, IR can occur in multiple key metabolic organs such as the liver and skeletal muscle. Nevertheless, insulin sensitivity and lipid metabolism may substantially differ between organs within an individual (59). Moreover, the extent to which IR is present in these distinct organs may vary among individuals. These inter-individual differences in tissue-specific insulin sensitivity may partly be explained by the location where excess lipids are stored. Ectopic fat and lipotoxic intermediates affect metabolism on a local tissue level, as described above. Thus, intrahepatocellular lipids (IHCL) and intramyocellular lipids (IMCL) are therefore often linked to the development of liver-IR and muscle-IR, respectively (5). Nonetheless, ectopic fat is not only related to site-specific disturbances in glucose homeostasis and IR, but also to more systemic disturbances (40). It could be speculated that tissue specific-IR may lead to a redistribution of substrates from the IR tissue toward other tissues during postprandial conditions (60). For example, muscle-IR may promote postprandial glucose supply to the liver, leading to increased *de novo* lipogenesis and hepatic steatosis (61, 62). This highlights the complex relationship between ectopic fat deposition and tissue-specific IR. Also, it should be noted that liver- and muscle-IR often coexist, which may bi-directionally affect metabolic perturbations in both organs. However, irrespective of the relationship between liver and skeletal muscle fat accumulation and liver- and muscle-IR, both IHCL and IMCL seem to play an important role in the progression toward T2DM (2). Clearly, multiple factors play a role in the complex relationship between ectopic fat deposition and tissue specific-IR, and careful interpretation of research findings is therefore needed.

Interestingly, plasma lipid profiles and lipid handling seem to differ between various IR-states, contributing to ectopic fat accumulation in both liver and skeletal muscle (33, 63). More specifically, higher fasting and postprandial plasma TAG, FFA, and VLDL-TAG concentrations have been observed in individuals with IGT as compared to impaired fasting glucose (IFG) (63–66), and in T2DM vs. non-diabetic individuals (67). Furthermore, an early postprandial increase in TAG concentration and higher skeletal muscle uptake TAG was observed in both IFG and IGT as compared to NGT (63). Additionally, IGT individuals showed higher skeletal muscle uptake of VLDL-TAG, higher intramuscular TAG content and higher intramuscular saturation of FFA compared to IFG individuals (33).

Interestingly, although IGT and IFG are both IR-states, these seem to differ in their tissue-specific IR, as liver-IR and muscle-IR seem to be the primary disorders in IFG and IGT, respectively (10, 68–70). Consistently, IGT is characterized by a decreased peripheral glucose disposal (71), whereas IFG is characterized by impaired insulin-induced suppression of liver gluconeogenesis and glycogenolysis (69). Not surprisingly, both IFG and IGT have been linked to the accumulation of ectopic fat in liver and skeletal muscle (72–74). Interestingly however, liver-IR is often considered to be a more severe IR-state as compared to muscle-IR. To exemplify, liver-IR seems to be accompanied with elevated postprandial levels of TAG and VLDL compared to muscle-IR (67), and has been linked to more severe cardiometabolic complications (75). This seems opposite to the finding that IGT is accompanied by higher fasting and postprandial plasma TAG and VLDL-TAG levels (63–66). These findings emphasize that the relationship between ectopic fat deposition, glucometabolic status (i.e., NGT, IFG, IGT, T2DM) and IR is complex, and suggest that these inter-relationships are dependent on certain exogenous (e.g., sex, ethnicity, and age) and/or endogenous factors (e.g., physical activity and diet). A better understanding of the different factors involved in these metabolic perturbations is needed to develop more personalized strategies to prevent or reverse IR, as discussed below.

### Endogenous and exogenous factors affect ectopic fat and liver- and muscle-IR

#### Endogenous factors

Sex-differences in health and disease is a well-recognized concept in literature (76–78). More specifically, sex differences have been linked to ectopic fat accumulation and IR (9). Difference between sexes may be partly related to differences in body fat distribution between men and women. Generally, women have a higher percentage of subcutaneous adipose tissue (SAT) as compared to BMI-matched men, who generally have more visceral adipose tissue (VAT) (79, 80). Interestingly, the expandability of subcutaneous adipose tissue seems to be a critical factor in the development of insulin resistance (81, 82). Lipids may be predominantly stored in SAT before marked VAT expansion occurs (81, 83). VAT has been linked to higher levels of inflammatory markers, insulin resistance and other cardiometabolic complications (83, 84). Moreover, in general women accumulate more adipose tissue in the gluteo-femoral as compared to the abdominal fat depot. Abdominal obesity is associated with an increased risk of developing type 2 diabetes and cardiovascular diseases (85). In contrast, lower body fat has protective properties that are associated with an improved cardiometabolic risk profile in men and women (86, 87). These metabolic differences in adipose tissue depots may partially explain why women are relatively protected against metabolic diseases compared to men with the same BMI (84).

Furthermore, although not completely elucidated, the metabolic differences between men and women are, at least partly, a consequence of differences in hormonal status. Estrogen has been shown to reduce IHCL accumulation and IR in both sexes (77). Until menopause, women have a lower risk for developing fatty liver, whereas post-menopausal women have a similar risk compared to age-matched males. The increased risk after menopause may be the result of the marked drop in estrogen concentrations during the menopausal phase (88). Indeed, estrogen treatment in the menopausal phase is protective for the development of non-alcoholic fatty liver (89). Furthermore, BMI was reported to be independently related to IHCL content in both men and women, whereas postprandial glucose level was independently related to IHCL content in women only (90). In contrast to IHCL content, some studies reported that IMCL content was significantly lower in both lean and overweight men as compared to BMI matched pre- and post-menopausal women (91–93), although conflicting data have also been reported (94). Noteworthy, for a given BMI, men generally have more muscle mass as compared to women, which may at least partly explain the latter findings (8). Interestingly, despite a higher IMCL content in women, insulin sensitivity and plasma lipid levels were similar between men and women, suggesting that women are relatively protected against lipid-induced IR (93). The relative protection against IMCL was, however, not observed in women using oral contraceptives (93). Interestingly, higher IMCL, lower fractional synthesis rate (FSR) and lower oxidative capacity was observed in prediabetic vs. NGT men, while no differences were observed in these parameters in prediabetes vs. NGT age and anthropometry matched post-menopausal obese women (95). Together, these findings suggest sex-specific differences in both IHCL and IMCL content. Whether these sex-specific differences in ectopic fat are dependent or independently related to IR, and which mechanisms are involved, is not yet fully understood and requires further investigation (59).

The relationship between ectopic fat storage, IR and sex seem to be largely dependent on the population. IMCL was significantly associated with IR in European Americans, while in African Americans, IMCL varied independent of IR (96). Another study reported a stronger association between IHCL and IR in individuals born in Iraq as compared to individuals born in Sweden (97). Moreover, it is well-established that South-Asians are more likely to develop IR and have more ectopic fat (both IHCL and IMCL) when compared to BMI-matched Caucasians (98–101). A possible explanation for this could be that South-Asians have a decreased lipid storage capacity in subcutaneous adipose tissue, leading to an excess flux of lipids toward other tissues such as visceral adipose tissue, the liver, pancreas, heart, and skeletal muscle (102). Interestingly, when IHCL and IMCL content in South-Asian and Caucasian men were adjusted for whole-body insulin sensitivity, IMCL was similar, while Asian men maintained a two-fold higher IHCL content, compared to Caucasian men (100). These findings suggest that the amount of IHCL, in contrast to IMCL content, varied independent of whole-body insulin sensitivity in South-Asians. In a study of Goedecke et al. (103), IHCL content was lower and IMCL content was higher in pre-menopausal black vs. white women. Interestingly, this same study reported that the association between ectopic fat (both IHCL and IMCL) and IR was only present in black women (103). Noteworthy, the total amount of lipids rather than the concentration of toxic lipid-intermediates was measured in these studies. Therefore, it remains uncertain whether the relationship between lipid-intermediates in liver and skeletal muscle and insulin sensitivity depends on ethnicity. Differences between ectopic fat and IR in these populations may be the result of certain genetic polymorphisms. For example, polymorphism of the ApoC3 and PNPLA3 gene have been linked to alterations in IHCL, and are more prevalent in South-Asian and Hispanic individuals, respectively (52, 104, 105). To conclude, the studies described above illustrate that the amount of ectopic fat may not always be directly related to IR, and seems to differ between populations.

Age seems to be a very relevant factor in the etiology and pathophysiology of IR, and seems to be positively related to ectopic fat accumulation (8, 106). Interestingly, there also appears to be an interaction between sex and age, especially related to the pre- or post-menopausal state of women, as already indicated above. More specific, pre-menopausal women are as insulin sensitive as age-matched men, despite higher IMCL content, while this relative protection against IR disappears after menopause (93). However, whether relationships between ectopic fat, IR, age and sex remain after adjustment for visceral fat or total fat mass is not fully known, but has been extensively reviewed elsewhere (8).

#### Exogenous factors

Lifestyle factors, such as unhealthy diet and a lack of physical activity, are key factors for the accumulation of ectopic fat and the development of IR (5, 10). Physical activity, with its different modalities such as resistance, endurance, or concurrent exercise, exerts several metabolic advantages. For example, improvements not only in cardiovascular fitness but also ectopic fat and insulin sensitivity are often observed (5). Furthermore, dietary intake is an important exogenous factor in relation to ectopic fat and IR. Hypocaloric diets, with resulting weight loss, will lead to a loss of body weight and fat mass, which is accompanied by a decrease in ectopic fat storage (5, 107, 108); metabolic improvements are often observed (5). In addition, the individual macronutrients in the diet, fats, carbohydrates, and proteins, seem to determine metabolic adaptations that have been related to both ectopic fat and IR. In the next section, we will focus on the relationship between macronutrient composition of the diet, ectopic fat storage, and IR.

## Macronutrient quality and quantity impact ectopic fat and IR

Ample data is available on the effect of different types of isocaloric diets with varying macronutrient composition on IR. Nevertheless, fewer studies are available that report the effects of a specific diet on both IR and ectopic fat accumulation. As discussed, the accumulation of ectopic fat is a major factor in the development and progression of IR. To select relevant studies, a semi-systematic literature search was performed. A literature search was performed in January 2018 using the databases PubMed and Google Scholar. The search strategy consisted of a combination of the following search terms using the Boolean operator “AND” and “OR”:

“Insulin resistance”[MeSH], “choristoma”[MeSH], “fatty liver”[MeSH], “Non-alcoholic Fatty Liver Disease”[MeSH], “liver steatosis” [TIAB], “ectopic fat”[TIAB], “intramuscular fat”[TIAB], “intramuscular adipose tissue”[TIAB], “muscle fat”[TIAB], “Glucose tolerance”[TIAB], “impaired fasting glucose”[TIAB], “impaired glucose tolerance”[TIAB], “energy metabolism”[Mesh], “metabolic flexibility”[TIAB], “liver insulin resistance”[TIAB], “hepatic insulin resistance”[TIAB], “muscle insulin resistance”[TIAB], “peripheral insulin resistance”[TIAB], “liver insulin sensitivity”[TIAB], “hepatic insulin sensitivity”[TIAB], “muscle insulin sensitivity”[TIAB], “peripheral insulin sensitivity”[TIAB], “dietary proteins”[Mesh], “dietary carbohydrates”[Mesh], “dietary fiber”[Mesh], “dietary fats”[Mesh], “diet therapy”[Mesh], “diet”[Mesh].

Articles included in Table 1 are selected with the following inclusion and exclusion criteria: articles written in both English and Dutch languages were included. Articles were excluded if *in vitro* or animal models were used. Studies examining the effects of single meals (acute setting) rather than dietary interventions, studies investigating hypo- or hyper-caloric diets, and review articles were excluded. Endnote X7 was used for the management and selection of articles. The search yielded a total of 729 papers. The majority of the studies were excluded based on abstract (*n* = 694), and others after having read the full-text (*n* = 25), resulting in a final selection of 10 original articles (Table 1).

**Table 1:** Effect of isocaloric diets on insulin resistance and liver and muscle fat content in populations at risk for T2DM.

**References**	**Diet [E%fat (F); E%carbohydrate (C); E%protein (P)]**	**Duration**	**Participants**	***N***	**% Females**	**Age (yrs)**	**IR measure**	**Statistical significance**	**Ectopic fat measure**	**Statistical significance**
(12)	SFA diet: [38F (SFA: 16); ?C; ?P] MUFA diet [38F (MUFA: 20); ?C; ?P] LF+HCC diet (28F; ?C; ?P) LF+HCC+1.24 g/d PUFA (28F; ?C; ?P)	12 weeks	Obese	24 26 16 18	50 50 50 50	56 58 57 58	HOMA-IR	N.S. N.S. N.S. N.S.	Skeletal muscle biopsy	Muscle: N.S. Muscle: N.S. Muscle: N.S. Muscle:−45%, *P* < 0.05
(109)	MED diet [44F (MUFA: 23F); 34C; 16P] LF + HCC diet [21F (MUFA: 8); 49C; 24P]	6 weeks	Obese NAFLD	12	50	55	*M-*value	−1.7, *P* < 0.01	MRS	Liver: −39%, *P* < 0.05 Liver: N.S.
(110)	HF + LC diet (55F; 30C; 15P)	3 weeks	Overweight	10	0	56	*M-*value	N.S.	MRS	Liver: +17%, *P* < 0.05 Muscle: N.S. Liver: −13%, *P* < 0.05 Muscle: N.S.
	LF + HC diet (20F; 65C; 15P)									
(111)	HF + LC diet (60F; 10C; 30P) LF+HC diet (30F; 50C; 20P)	4 weeks	Overweight	11 11	100 100	31 32	HOMA-IR	N.S. N.S.	MRS	Muscle: N.S. Muscle: + 49%, *P* < 0.05
(115)	HP + MUFA [40F (MUFA: 20.5); 30C; 30P]	5 weeks	Obese	10	100	?	HOMA-IR	−0.74, *P* < 0.01	MRS	Liver: −49%, *P* < 0.01 Muscle: N.S.
(116)	DG diet (30F; 50C; 20P) DG+n-3 diet (1 g/d PUFA) (30F; 50C; 20P)	6 months	Obese NAFLD	16 16	56 39	51 50	HOMA-IR	N.S. - 0.73, *P* < 0.05	Abdominal ultrasound	Liver: N.S. Liver: −25%, *P* < 0.01
(121)	Low-GI+LF diet, GI < 55 [23F (SFA: 7); 57C; 17P]	4 weeks	Overweight	20	65	69	Matsuda HOMA-IR	0.6, *P* < 0.05 N.S.	MRS	Liver: −2.2%, *P* < 0.01
	High-GI + HF diet, GI > 70 [43F (SFA: 24); 38C; 16P]			15	60	69	Matsuda HOMA-IR	N.S. N.S.		Liver: N.S.
(130)	60 g/d whey supplementation	4 weeks	Obese	11	100	38	HOMA-IR	N.S.	MRS	Liver: −21%, *P* < 0.05
(13)	HP(plant) diet: (30F; 40C; 30P) HP(animal) diet: (30F; 40C; 30P)	6 weeks	Obese T2DM	19 18	37 33	64 65	AT-IR	−0.37, *P* < 0.05 − 3.38, *P* < 0.05	MRS	Liver: −36%, *P* < 0.001 Liver: −48%, *P* < 0.001
(14)	MUFA diet [46F (22 MUFA); 40C; 14P]		15	47		62		N.S		Liver: -17%, P < 0.001
	High fiber diet (20g fiber/100kcal) (28F	15	53		63		N.S.			Liver: N.S
	55C; 17P)	12 weeks	Obese			DI		MRS
	DG diet (34F; 49C; 17P)			13	23	60		N.S		Liver: N.S

IR, insulin resistance; IS, insulin sensitivity; DI, disposition index; HOMA-IR, Homeostatic model assessment insulin resistance; AT-IR, adipose tissue IR (fasting plasma glucose *fasting free fatty acids); LF, low fat; HF, high fat; MUFA, mono-unsaturated fatty acid; n-3, omega 3 fatty acid; SFA, saturated fatty acid; LC, low carbohydrate; H(C)C, high (complex) carbohydrate; HP, high protein; GI, glycemic index; MED, Mediterranean; DG, dietary guidelines; MRS, magnetic resonance spectroscopy; N.S., not significant.

### Carbohydrate/fat ratio of the diet

Four different studies included in Table 1 investigated the effect of a low-fat high-complex carbohydrate (LFHCC) diet on ectopic fat and IR in overweight or obese individuals. The studies investigated the effect of diets that were slightly different in macronutrient composition and study duration (ranging between 3 and 12 weeks; Table 1). However, these studies consistently reported no significant effects of a LFHCC or a low-fat high- carbohydrate (LFHC) diet on IR, determined by either HOMA-IR or the M-value (12, 109–111). Study outcomes were less consistent with respect to ectopic fat content. The study of Van Herpen et al. (110) reported a significant decrease in IHCL following 3 weeks of a LFHC diet [21 energy percent (E%) fat, 49 E% carbohydrate, 24 E% protein], while Ryan et al. (109) did not report changes in IHCL after a 6 week LFHCC diet (20 E% fat, 65 E% carbohydrate, 15 E% protein). Two studies including LFHC or LFHCC diets did not report changes in IMCL in overweight and obese individuals after 3 or 12 weeks of dietary intervention (12, 110), while the study by Parente et al. (111) showed a 49% increase in IMCL content after a 4-week LFHC diet. It was not clear whether the carbohydrates were mainly complex or not. These discrepant findings may partly be explained by differences in the metabolic phenotype of the study participants. For example, it has been found that the intake of a carbohydrate-rich meal resulted in more than two–fold increase in *de novo* lipogenesis and TAG synthesis leading to a significant increase in IHCL content in the liver in IR individuals as compared to insulin sensitive individuals (61). Interestingly, in the latter study, no changes were observed in IMCL content between the groups. However, the proportion of saturated (SFA), mono-unsaturated (MUFA), and poly-unsaturated (PUFA) fatty acids in these diets was either not comparable or not described, making it difficult to compare these studies.

### Dietary fat quality

There is substantial evidence that dietary fat quality is related to ectopic fat storage. In an *in vivo* study in rats and an *in vitro* study in myotubes, it was reported that SFA preferentially accumulate as DAG in skeletal muscle and may thereby potentially interfere with insulin signaling, whereas MUFA and PUFA more readily convert to TAG (112, 113). Also, after incubation of human skeletal muscle cells with either the MUFA oleic acid or the SFA palmitic acid, higher lipolytic rates were observed with oleic acid (114). Furthermore, the glucose and insulin responses to a high SFA meal was greater when compared to a high PUFA meal in IR men, indicating an impaired postprandial insulin sensitivity, which was accompanied by a decreased skeletal muscle lipid turnover and FSR of TAG and DAG after the high SFA meal (11).

These data are supported by longer-term human dietary intervention studies. The study of Jans et al. (12) reported no significant change in IMCL following a 12-week LFHCC diet in obese older adults, but reported a significant reduction in IMCL when this diet was supplemented with 1.24 g/day of PUFA omega-3 (LFHCC+n3 diet, Table 1). Interestingly, the latter study also reported that a 12-week diet rich in MUFA, defined as 20E%, had the tendency to decrease skeletal muscle FSR into DAG and TAG compared to pre-intervention (12). Both the MUFA diet and LFHCC+n-3 diet decreased the expression of lipogenic genes, although this was not reflected in IMCL changes in participants after completing the MUFA diet (12). Nonetheless, it shows that the LFHCC+n3 and MUFA diets have the potential to decrease skeletal muscle lipid (intermediate) accumulation, which in turn may affect insulin signaling. However, neither of the diets showed that this effect was related to changes in IR (12).

Clearly, dietary fat quality may have significant effects on ectopic fat and potentially also on insulin sensitivity. Significant reductions in IHCL content and improvements in insulin sensitivity were found following a MUFA diet (5 weeks) (115), a diet supplemented with omega-3 (6 months) (116) and a Mediterranean diet (high in MUFA and PUFA) (6 weeks) (109), see also Table 1. Interestingly however, excessive intake of fat (>55E% for 3 weeks) in the diet (including high PUFA and MUFA) resulted in a 17% increase in IHCL content in overweight men, but did not change IMCL (110). Whether these changes in IHCL content are also related to changes in insulin sensitivity remains to be elucidated. Several longer-term (12–24 weeks) large dietary intervention studies that investigated the effects of isoenergetic replacement of SFA with MUFA and/or PUFA on insulin sensitivity either did not report significant improvements in insulin sensitivity following the different diets (117, 118), or only reported significance when absolute intake of fat was not high (< 37E%) (119).

### Carbohydrate quality

The metabolic implications of dietary carbohydrates are largely dependent on the type of carbohydrate. The intake of simple carbohydrates have especially been linked to metabolic disorders (120). A diet low in glycemic index (GI), which is a relative ranking of foods and their effect on blood glucose values, may therefore be beneficial in relation to insulin sensitivity, and has also been linked to decreased IHCL accumulation (120). Indeed, an isocaloric low-GI diet for 4 weeks in overweight older adults led to a significant decrease in IHCL and improvement in insulin sensitivity (Matsuda index) compared to a 4-week isocaloric high-GI diet (121) (Table 1).

Individual carbohydrates and their effect on insulin sensitivity and ectopic fat have been studied. For example, excessive dietary fructose, as present in sugar-sweetened beverages, is thought to promote hepatic *de novo* lipogenesis, IHCL and IMCL content, and has accordingly been correlated to the development of cardiometabolic diseases (122–124), although This has been confirmed in animal models (125, 126). However, a 4-week isocaloric high-fructose diet (1.5 g/kg/body weight) in healthy men resulted in an increase in fasting plasma concentrations of TAG, VLDL-TAG, and glucose, but did not significantly alter ectopic fat content and insulin sensitivity in the liver and skeletal muscle (127). Furthermore, another study measured fat content in liver or skeletal muscle by computed tomography (CT) and found no differences between a 10-week isocaloric diet high in fructose and an isocaloric diet high in sucrose (123). Unfortunately, insulin sensitivity was not reported in the latter study. Finally, a study including both NGT as well as IGT individuals compared the effect of 50 g of added carbohydrate to the diet from either honey, sucrose, or high-fructose for 2 weeks (128). Insulin sensitivity and lipid levels were not different between the different types of carbohydrates after 2 weeks of dietary intervention (128). Although the complexity of dietary carbohydrates may be important in relation to insulin sensitivity and ectopic fat, simple carbohydrates, such as fructose and sucrose, when consumed in iso-energetic exchange for other CHO sources may not be directly related to differences in insulin sensitivity and ectopic fat (129).

### Protein intake

The amount of protein in the diet also seems to be important in relation to IR and ectopic fat. Markova et al. (13) showed that a 6-week diet high in protein (30 E%, either high in plant- or animal-source protein) reduced IHCL content in individuals with T2DM, as measured by MRS. Remarkably, these diets induced pronounced changes in IHCL, ranging from approximately −36% for the plant protein diet to −48% for the animal protein diet. Additionally, the reduction in IHCL content correlated with several parameters related to glucose homeostasis, including decreased fasting glucose levels, improved whole-body insulin sensitivity and decreased *de novo* lipogenesis. A decreased IHCL content following a high protein diet was also reported in a study supplementing whey protein (60 g/d) for 4 weeks in obese individuals (130). However, the latter study was not controlled. The effect on IMCL was not reported for either of the above-mentioned studies. The number of studies describing the effect of protein quality and quantity is limited; however, it seems that protein quality and quantity in the diet may have a significant effect on ectopic fat accumulation in the liver.

Overall, drawing conclusions on diets that are most effective in improving ectopic fat is highly challenging. Comparing dietary intervention studies is very difficult as characteristics between populations vary with age, sex, BMI, amongst others. Moreover, the duration of the intervention and macronutrient composition between studies differs considerably. Also, the majority of studies measure either IHCL or IMCL, but rarely do they assess both. Moreover, measures of IR (i.e., HOMA-IR, Matsuda Index, M-value) differ between studies and may reflect different etiologies of IR (131, 132). In addition, lipid-intermediates were not assessed in most of these studies, and the majority of the studies performed to date did not take liver- and muscle-IR into account when assessing the effectiveness of interventions. Finally, the baseline metabolic phenotype of individuals may be an important determinant on the effectiveness of dietary interventions, as discussed in more detail in the next section.

## Differential effect of macronutrients on ectopic fat in distinct IR phenotypes; toward personalized nutrition

For several decades, many studies have focused on diets either low in carbohydrate or in fat as the most effective approach to improve glucose homeostasis and manage T2DM (133). Interestingly, however, not all individuals may benefit from the same diet. Recent studies have shown large inter-individual variation in response to a meal (134), which may also explain why individuals respond differently to the same dietary intervention. Indeed, in recent years, several studies have demonstrated that the macronutrient quality and quantity in the diet can lead to a differential response, depending on the individual's metabolic phenotype (11, 12, 14, 75, 135, 136). More specifically, this response may be dependent on liver- or muscle-IR, ectopic fat content, age, ethnicity, sex, and likely other factors that have not been studied extensively thus far.

There has been a debate on different dietary approaches in the prevention of T2DM, one is the Mediterranean diet, rich in olive oil, which may provide cardiovascular benefits. Second, diets low in fat and high in complex carbohydrates with increased fiber content (within the context of a lifestyle intervention) may decrease the cumulative incidence of diabetes by more than 50% over 3–6 years (137–139). In the CORDIOPREV-DIAB study, the Mediterranean (high in MUFAs) and the LFHCC dietary patterns were compared with respect to tissue-specific insulin resistance and beta-cell function in cardiovascular patients not treated for diabetes (*n* = 642, analysis at baseline and at 2 years follow-up) (75). Interestingly, the change in disposition index (DI), which is a composite score of insulin sensitivity and secretion, after a long-term dietary intervention, was related to the tissue-specific IR phenotype of the participants at baseline. The tissue-specific IR phenotypes were defined as no-IR, muscle-IR, liver-IR or combined muscle and liver-IR, based on tertiles of the muscle insulin sensitivity index (MISI) and hepatic insulin resistance index (HIRI), which was modeled from an OGTT (140). Interestingly, the Mediterranean diet was reported to be most beneficial for individuals with muscle-IR, while the LFHCC diet was most beneficial for the liver-IR phenotype (75). In addition, a recent *post-hoc* analysis in the European project LIPGENE, which focused on the effects of dietary fat quantity and quality in the metabolic syndrome (MetS), found that IR MetS individuals were more sensitive to health effects from the substitution of a high-saturated fat diet by either high-MUFA or high (complex)-carbohydrates (with added n-3 PUFA) diets (15). On the contrary, the individuals with higher insulin sensitivity were more susceptible to the detrimental effects of SFA. Furthermore, Guess et al. (141) recently reported that 6 weeks of supplementation with the dietary fiber inulin (30 g/d) decreased HOMA-IR in IFG but not in IGT individuals. These results may indicate that inulin has beneficial effects in subjects with liver-IR rather than muscle-IR, as IFG individuals have often a more pronounced liver-IR.

**Figure 1:**
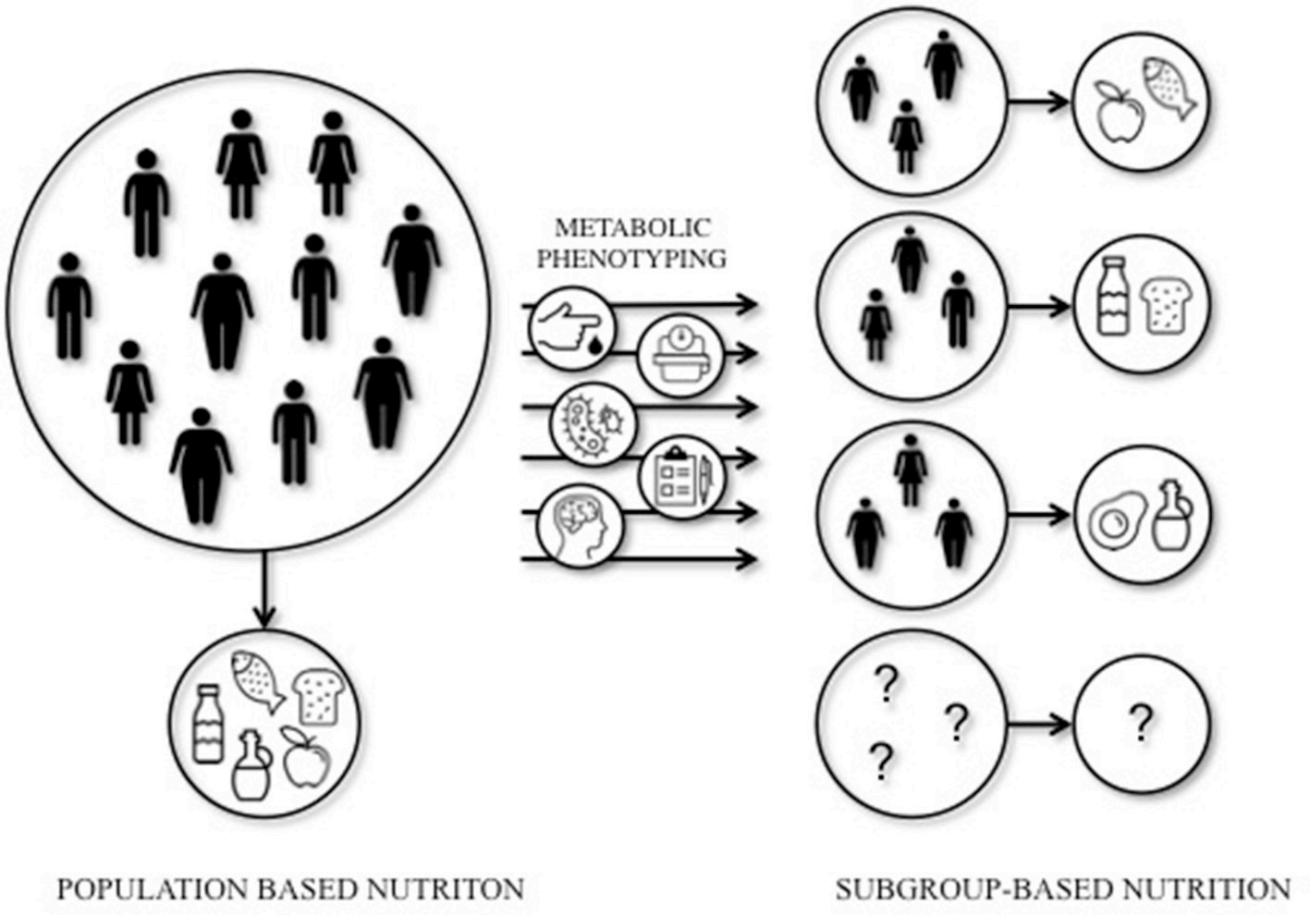
Population-based vs. subgroup-based nutrition. Large inter-individual variation can be seen in the response to dietary interventions. This implies that population-based nutritional interventions may not always lead to the most optimal (metabolic) outcomes for each individual. A subgroup-based approach that integrates among others cardiometabolic parameters, anthropometrics, gut microbiota, mental status, age, sex, and ethnicity, may increase the efficacy of dietary interventions. Future studies should elucidate the most optimal diet for a certain (metabolic) phenotype.

Mediterranean or LFHC high-fiber diets may be healthy for all, however, these studies indicate that dietary prevention or treatment may require a more subgroup-based or personalized approach to optimize the effects of dietary interventions. Indeed, a recent study by Zeevi et al. (134) demonstrated that personalized diets seem more effective in lowering postprandial blood glucose responses compared to diets based on expert advice. In this study, a machine-learning approach was used to predict blood glucose responses, using parameters such as dietary habits, physical activity, gut microbiota composition, and anthropometrics, thereby developing the most optimal diet for each individual. The studies that are mentioned in this section are illustrative of the importance of personalized nutrition to optimize the effect of dietary interventions in IR individuals. Targeting tissue-specific IR phenotypes, such as liver- and muscle-IR, might prove to be an effective strategy to optimize metabolic outcomes of dietary interventions (Figure 1).

Nonetheless, the available literature to date does not provide sufficient evidence from prospective studies into the optimal quantity and quality of macronutrients in the diet based on metabolic phenotype. The tissue-specific IR phenotypes are related to a variety of different characteristics, such as ectopic fat, glucose control, gut microbiota, sex, age, and others, and are likely not isolated entities. Defining metabolic phenotypes is difficult and may therefore not always do justice to its complexity. Moreover, although not surprising, the studies mentioned in this review only focus on a specific phenotype, such as muscle- vs. liver-IR or mild- vs. severe-IR, and mainly focus on Caucasian, middle-aged populations. Therefore trying to understand an optimal diet for other ethnicities, age groups, or other metabolic (sub-)phenotypes such as IFG vs. IGT is difficult. Recommendations or conclusions on the optimization of diets for each metabolic phenotype are therefore not yet available (Figure 1).

Available evidence clearly indicates that there is great potential to optimize the effectiveness of dietary interventions on glucose homeostasis by, for example, targeting liver- and muscle-IR phenotypes. A first step toward the development of more personalized nutrition could be to investigate the role of tissue-specific IR and related ectopic fat content in the effectiveness of dietary interventions, in particular when studying the effect of manipulation of the macronutrient composition of the diet. Macronutrient composition, both quality and quality, is highly important and should be taken into account for these types of intervention studies. To develop effective personalized dietary interventions, it seems necessary to perform extensive phenotyping of individuals to understand the complexity of metabolic regulation and its target for different macronutrients, as illustrated in Figure 1. Here, a schematic overview is given of the concept of subgroup-based or more personalized nutrition. Based on the baseline metabolic phenotype, a specific dietary intervention may be initiated to optimize intervention outcomes.

In conclusion, to understand the complexity of the interaction of an individual's metabolic phenotype and the response to diet, a detailed physiological phenotyping including the use of advanced -omics methodologies like (epi)genomics, metagenomics, and metabolomics is required. Additionally, mobile apps and wearable devices may facilitate real-time assessment of dietary intake and physical activity, and may provide individual feedback to optimize personalization of advices. By integrating these technologies with big data analytics, personalized nutrition has the potential to provide targeted nutrition and lifestyle guidance for more effective prevention and management of type 2 diabetes and related chronic diseases. Despite all technological advances, the step toward implementation into a public health and clinical setting is still more remote and also includes other factors like dietary preferences, the socio-economic context and behavioral factors. Evidence is required to demonstrate the efficacy, cost-effectiveness as well as additional benefits of a personalized or subgroup-based approach beyond a traditional approach before these nutritional interventions can be implemented in daily practice in the future.

## Author contributions

IT wrote the manuscript; SB, GG, and EB conceptualized, reviewed, and edited the manuscript. All authors have read and approved the final version of the manuscript.

### Conflict of interest statement

The authors declare that the research was conducted in the absence of any commercial or financial relationships that could be construed as a potential conflict of interest.
